# Influence of Post-Printing Polymerization Time on the Elution of Residual Monomers and Water Sorption of 3D-Printed Resin Composite

**DOI:** 10.3390/ma18122905

**Published:** 2025-06-19

**Authors:** Shaima Alharbi, Abdulrahman Alshabib, Hamad Algamaiah, Muath Aldosari, Abdullah Alayad

**Affiliations:** 1 Department of Restorative Dental Science, College of Dentistry, King Saud University, Riyadh 11362, Saudi Arabia; abdalshabib@ksu.edu.sa (A.A.); haljamaiah@ksu.edu.sa (H.A.); alayad@ksu.edu.sa (A.A.); 2 Department of Conservative Dentistry, College of Dentistry, Aljouf University, Sakaka 72388, Saudi Arabia; 3 Periodontics and Community Dentistry, College of Dentistry, King Saud University, Riyadh 11362, Saudi Arabia; amuath@ksu.edu.sa; 4 Oral Health Policy and Epidemiology, Harvard School of Dental Medicine, Boston, MA 02115, USA

**Keywords:** monomer elution, post-curing time, HPLC, water sorption, 3D resin composite, monomer release

## Abstract

This study evaluated the effect of post-printing polymerization time on residual monomer elution and water sorption in a 3D-printed resin composite. Eighty samples were fabricated and assigned to four groups based on post-curing duration: 0, 20, 40, and 60 min. Each group was subdivided according to two storage conditions (distilled water and 75% ethanol–water solution), and evaluated at 1 and 7 days. High-performance liquid chromatography (HPLC) quantified eluted monomers. Additionally, 40 specimens underwent a 4-month sorption/desorption cycle for water sorption and solubility assessment. Data were statistically analyzed using kernel regression (monomer data) and Welch ANOVA (water sorption and solubility) at a significance level of *p* < 0.05. BisEMA was the only monomer detected, with significantly higher elution recorded in ethanol-based storage. Increasing post-curing time notably reduced both monomer release and water sorption/solubility (*p* < 0.001); however, the optimal results were observed at 40 min post-curing. These findings suggest that extending post-curing beyond an optimal threshold does not further improve composite properties, underscoring the importance of identifying precise curing parameters in order to enhance durability and material performance.

## 1. Introduction

Dental composite materials are widely used in today’s dentistry because of their aesthetic properties and ability to bond effectively to tooth structures. Resin-based restorative materials are composed of an organic polymer matrix and inorganic filler particles dispersed within the matrix [1]. The most common resins used in the formulations of restorative composites are based on methacrylate monomers, such as Bis-GMA (bisphenol-A-glycidyl methacrylate), Bis-EMA (bisphenol A diglycidyl methacrylate ethoxylated), TEGDMA (triethyleneglycol dimethacrylate), and UDMA (urethane dimethacrylate) [2].

From a biological perspective, dental biomaterials designed to interact with living tissues should particularly be validated for their biocompatibility [3]. Biocompatibility is formally defined as the ability of a given material to evoke an appropriate biological response in the surrounding living tissue in the oral cavity [4]. In the case of methacrylate-based composites, the elution of unpolymerized monomers can potentially induce adverse biological responses, such as pulpal damage, allergic reactions, and mucosal irritations [5]. This underscores the importance of an efficient polymerization reaction that minimizes the elution of residual monomers [6]. In addition, the chemical and dimensional stability of dental resin composites is closely related to their water sorption and solubility [7]. Water absorption can lead to hydrolytic degradation, which compromises the mechanical integrity of the resin, while high solubility may increase the leaching of degradation by-products [8].

As for the relatively new technology of 3D additive-manufactured composites, resin-based materials used in the fabrication of splint devices and denture bases were shown to be more susceptible to water sorption than their milled, or conventional, counterparts [9,10,11,12]. The prolonged post-curing periods of 3D-printed dental resins significantly boosts their degree of conversion, thereby reducing the amounts of residual monomers available to leach out [13]. Studies have shown that extending post-curing time (up to around 20 min) markedly decreases monomer release and material solubility, while also lowering water sorption, ultimately yielding 3D resin properties and biocompatibility comparable to conventional cured controls [14]. However, most published work to date has focused on photopolymer resins used for short-term applications, such as surgical guides, splints, or interim restorations, rather than on the newer materials intended for permanent crowns and inlays [15,16,17]. Studies specifically evaluating these permanent crown resins remain limited in the literature [18].

Therefore, this study aimed to investigate residual monomer release using the water sorption and solubility of a currently used 3D resin material after post-polymerization periods with four different post-cure times. The null hypothesis was that 3D resin specimens with different post-curing times would show no significant differences in monomer elution, water sorption, and solubility.

## 2. Materials and Methods

### 2.1. Elution of Residual Monomers

#### 2.1.1. Specimen Preparation

**Figure A1:**
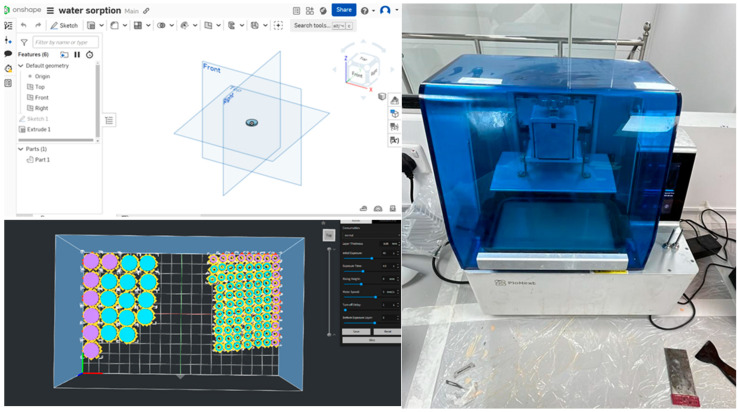
Digital design and 3D printing of composite specimens. CAD software (Onshape) was used to design standardized specimens for the water sorption and monomer release; these designs were exported as STL files and arranged efficiently on the printer platform with preset printing parameters. Specimens were subsequently printed using a Digital Light Processing (DLP) 3D printer.

**Figure A2:**
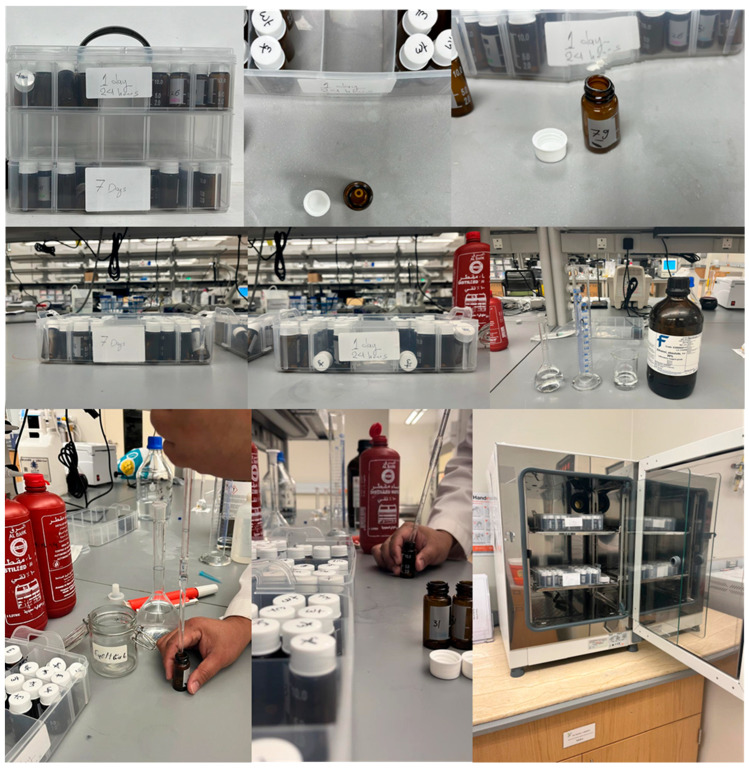
Workflow of sample preparation and monomer elution analysis. Samples of 3D-printed resin composites were labeled, sorted according to storage duration (1 day and 7 days), and immersed in specified media (distilled water or ethanol–water solution). Precise measurement and dispensing of storage media into glass vials were conducted using volumetric pipettes under controlled laboratory conditions. Finally, specimens were sealed and stored in an incubator maintained at 37 °C to simulate physiological conditions.

Eighty specimens were printed, using a 3D printer with a 3D permanent crown resin (Table 1). Printing parameters were set, in accord with the manufacturer’s recommendations, to a laser wavelength of 405 nm and a layer thickness of 50 μm. The samples were designed using the Onshape© CAD software (https://www.onshape.com, accessed on 1 March 2023, Seaport Boulevard, Boston, MA, USA), and exported in the stereolithography (STL) file format, with a hollow cylindrical shape with a 7 mm external diameter, 4 mm internal diameter, and 2 mm height, following ISO 10993-13 [19] (Figure A1). The specimens were washed using clean isopropyl alcohol for 3 min to remove residual uncured resin. The length, width, and thickness of each specimen was confirmed by measuring its dimensions three times with a high-precision digital caliper (Neiko 01407A Electronic Digital Caliper, Zhejiang Kangle Group, Wenzhou, China) with an accuracy of 0.02 mm. The post-curing process was performed using post-curing equipment (Shenzhen PioCreat 3D Technology, Shenzhen, China) with a UV intensity of 220 μW/cm^2^ and an internal temperature of 60 °C (140 °F). The samples were placed flat on a tray, approximately 5 cm between the light source and the samples, in such a manner that the upper surfaces of the samples were directly exposed to UV light. Afterward, the specimens were randomly divided, using research randomizer software (Urbaniak and Scott Plous, Lancaster, PA, USA), into four groups according to post-printing polymerization time. This included the green-state group (without post-curing) and groups associated with 20, 40, and 60 min post-curing times (n = 5). Two storage media were used: 75 vol.% ethanol/water, and distilled water. Each sample was placed in a 10 mL dark glass vial with 2 mL storage media and stored at 37 °C in an incubator. The collected storage solutions from each storage period were placed in vials to identify and quantify the eluted monomer using high-performance liquid chromatography (HPLC) analysis (Figure A2).

#### 2.1.2. Identification and Quantification of Eluted Monomers

For the characterization of the extracted residual monomers, reference standards for dental monomers were purchased (Table 2). HPLC with a photodiode array (PDA) detector was used (The i-Series HPLC and UHPLC systems, Shimadzu Scientific Instruments Incorporated, Kyoto, Japan). The gradient flow was set at a rate of 0.5 mL/min, with PDA detection at a 215 nm wavelength. The column temperature was set at 30 °C with a run time of 30 min. To quantify the eluted monomers in various media, a series of calibration curves was obtained by weighing 0.1 g of each of the four standards of interest separately (i.e., TEGDMA, UDMA, BisGMA, and BisEMA). Each standard was transferred into a 100 mL volumetric flask. The volumetric flask was used to measure the total solution volume precisely. The flask was shaken and then sonicated (ultrasonically agitated) for 10 min to ensure that the standards dissolved completely in the solvent. After sonication, more diluent (75% ethanol) was added until the total volume reached the 100 mL mark on the volumetric flask. The eluted monomers from the 3D samples were identified by comparing the retention time of each peak with that of the injected reference standard within the same chromatographic parameters (same mobile and stationary phases). The chromatographic data were collected and processed using the LabSolutions™ CL software (https://www.shimadzu.com/an/products/software-informatics/labsolutions-series/index.html, accessed on 19 June 2024, Shimadzu Scientific Instruments Incorporated, Kyoto, Japan).

### 2.2. Water Sorption and Solubility

**Figure A3:**
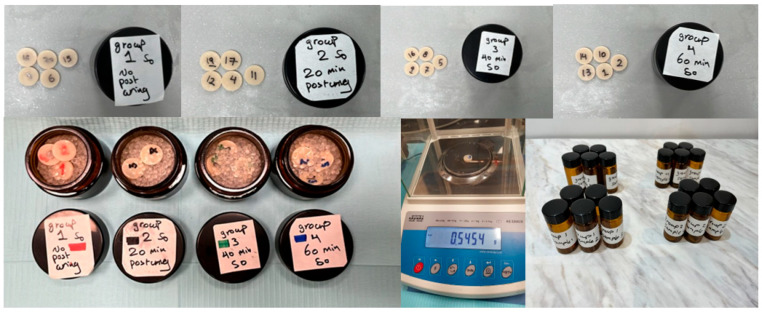
Sample preparation and water sorption analysis workflow. The 3D-printed composite samples were sorted into groups according to post-curing times (0, 20, 40, and 60 min). Samples were stored in airtight containers filled with silica gel to desiccate them and weighed using a high-precision analytical balance. After baseline measurements, samples were placed into sealed amber bottles filled with distilled water for designated storage durations to evaluate water sorption under standardized conditions.

Twenty specimens were fabricated using a 3D printing technique (n = 5) for the water sorption and solubility tests. The specimen design, 3D printing, washing, dimensional verification, and post-curing were performed as described above. After the samples were randomly assigned to the study groups of 0, 20, 40, and 60 min post-curing, they were placed in a desiccator containing silica gel at 37 °C ± 1 °C. After 24 h, each specimen was weighed using an analytical balance (Explorer™ Analytical, Ohaus Corporation, Parsippany, NJ, USA). This cycle was repeated until a constant mass (m1) was achieved, at which the mass loss of the specimens was ≥0.2 mg over 24 h. For thickness measurements, a digital caliper (Neiko 01407A Electronic Digital Caliper, Zhejiang Kangle Group, Wenzhou, China) was used to take two height measurements (Figure A3). The volume (V) of the specimen was then calculated in cubic millimeters using the following formula:

ν = πr^2^t,

where π = 3.14, r is the radius of the cross-section, and t is the thickness of the specimen.

All specimens were submerged in 10 mL of distilled water in separate dark glass vials for water sorption measurement. The vials were stored at 37 °C for 1, 2, 3, 4, 5, 6, 7, 14, 21, 28, and 56 days. The recorded weight of each was denoted as (m2). The specimens were then returned to aqueous storage, which was replenished weekly, maintaining the total volume of water at 10 mL.

For solubility measurement, the specimens were dried for 1, 2, 3, 4, 5, 6, 7, 14, 21, 28, 56, 80, and 84 days after completing the sorption cycle. Once the weight loss was no more than 0.2 mg, the constant final mass (m3) was recorded.

The weight increase (Wi%), water sorption (WSo), and solubility (Sol) were calculated using the following formulas:

Wi (%) = (m2 − m1)/m1 × 100 Wso (µg/mm^3^) = (m2 − m3)/V Sol (µg/mm^3^) = (m1 − m3)/V

where m1 is the mass before immersion in water, m2 is the mass after 56 days of immersion, m3 is the mass after desorption, and V is the volume of the specimen.

### 2.3. Statistical Analyses

The data collected were analyzed using statistical software (Stata BE, Version 18). A power analysis revealed that a sample size of 5 per group was found to meet the constraints of α = 0.05, effect size F = 0.6, and power = 0.95. Descriptive statistical analyses regarding the means, standard deviations, and percentages were performed. Statistical test assumptions were then tested using the Shapiro–Wilk test and scatter plots. The impacts of the post-cure times on monomer release under different storage conditions were examined using kernel logistic regression. The influence of post-printing polymerization time on water sorption and solubility was tested using Welch ANOVA.

## 3. Results

### 3.1. Monomer Release

**Figure 1:**
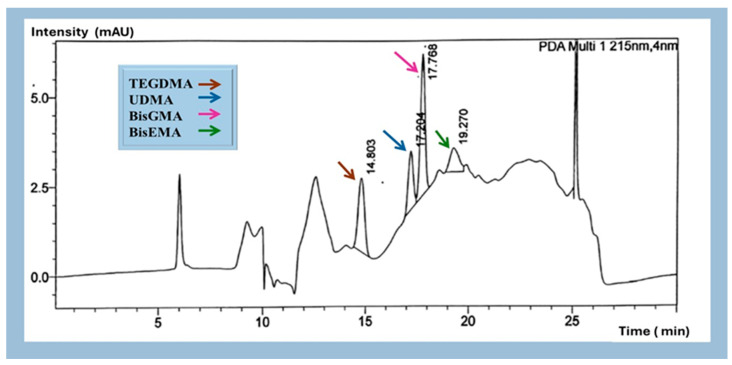
Identified chromatographic peaks of standard monomers.

**Figure 2:**
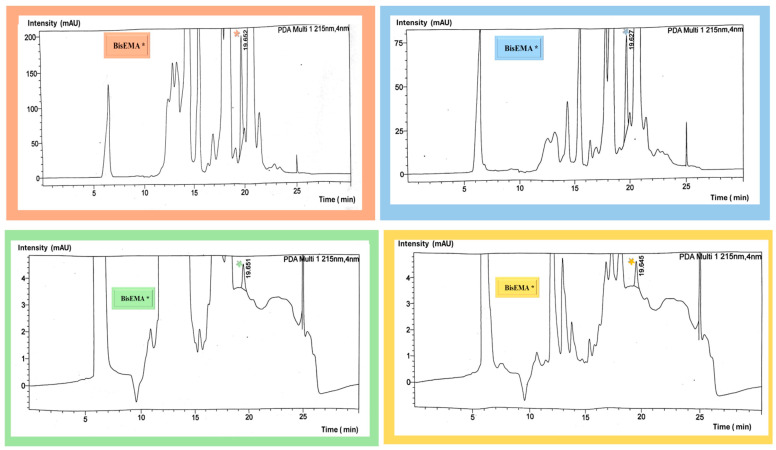
Chromatographic peaks detected by HPLC analysis of a representative sample from each group: no post-cure (orange), 20 min post-cure (blue), 40 min post-cure (green), and 60 min post-cure (yellow). The star (*) indicates the BisEMA peak, detected across all the groups at the same retention time of 19.6.

**Figure 3:**
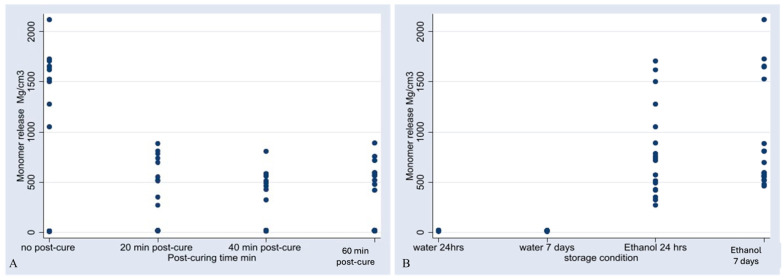
Scatter plots of the relations between post curing time (**A**) and storage condition (**B**) with respect to monomer release.

**Figure A4:**
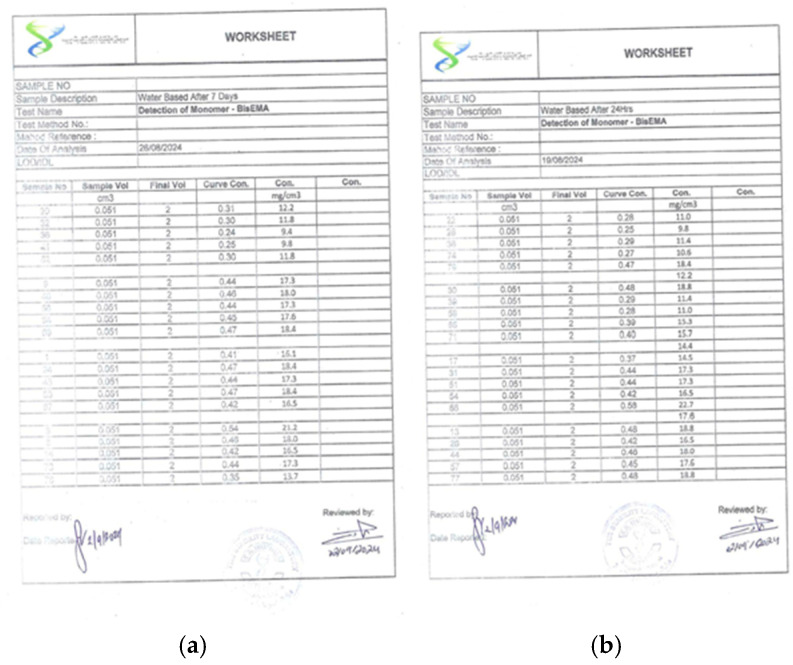

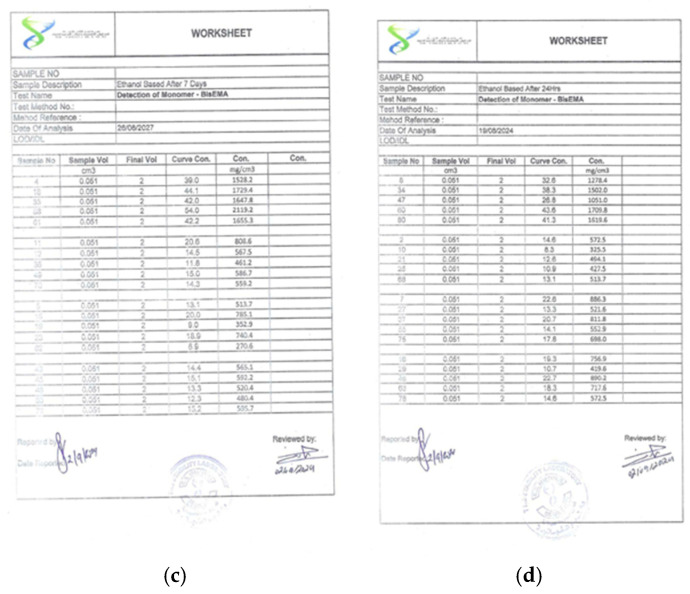
Raw data sheets from high-performance liquid chromatography (HPLC) analysis for BisEMA monomer elution from 3D-printed resin composite. Each worksheet displays the raw output, including sample volumes and curve concentrations, and the resulting calculated concentrations of BisEMA in µg/mL. These unprocessed data served as the primary bases for subsequent statistical analysis. (**a**) Water-based storage after 7 days. (**b**) Water-based storage after 24 h. (**c**) Ethanol-based storage after 7 days. (**d**) Ethanol-based storage after 24 h.

The respective retention times and absorbance intensities of identified monomers are presented in Figure 1. BisEMA monomer was detected in the tested 3D composite material after the analysis of the study samples, as the retention times and peak areas matched the standard (Figure 2 and Figure A4). The data normality assumption was tested using the Shapiro–Wilk test, which showed significant deviation from the normal distribution (*p* < 0.001). The results of the linearity tests using scatter plots showed nonlinear relationships between post-curing time and the concentration of eluted monomers (Figure 3A) and between the storage conditions and monomer elution (Figure 3B). Therefore, the effects of the post-curing times and storage conditions on monomer elution were statistically analyzed using a kernel regression model (Table 3). The model demonstrated a strong fit, with R2 value of 0.9535, indicating that 95% of the variability in the concentration of the eluted monomers was explained by the post-cure times and storage conditions. The mean concentration observed was 425.36 (95% confidence interval [CI]: 304.0363–548.1826), and this estimate was statistically significant (z = 7.12, *p* < 0.01).

The nonparametric regression analysis revealed that post-curing significantly decreased the monomer concentration (*p* < 0.01). The negative coefficients from the post-curing time comparisons indicate that increased post-curing duration results in lower monomer release, relative to a lack of post-curing. Specifically, the eluted monomer concentrations decreased by 478.95, 519.96, and 480.15 units after 20, 40, and 60 min of post-curing, respectively (all *p* < 0.01). The 40 min post-curing group exhibited the least-eluted monomers, with concentrations decreasing by 519.96 units on average compared with those in the no post-curing group (95% CI: 661.6359–365.1328).

**Figure 4:**
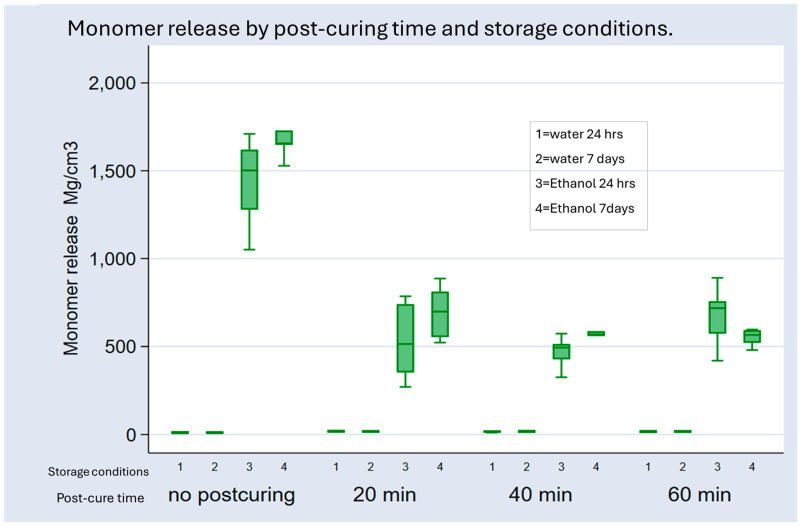
Box plots describing the effects of variations in post-printing polymerization time and storage conditions on monomer release.

Storage in ethanol for 24 h or 7 days led to a statistically significant increase in monomer release, compared with storage in water for 24 h (*p* < 0.001). Storage in ethanol for 7 days resulted in the highest elution of monomers (β = 872.49, *p* < 0.001). Storing the samples in water for 7 days increased monomer release by 0.27 units on average compared with storing them in water for 24 h (95% CI: 8.618262–15.89887). While this point estimate suggests a slight increase in concentration with longer water storage, this increase was not statistically significant (*p* > 0.98). The most considerable monomer release occurred from the non-cured samples stored for 7 days in ethanol (Figure 4).

### 3.2. Water Sorption and Solubility

**Figure 5:**
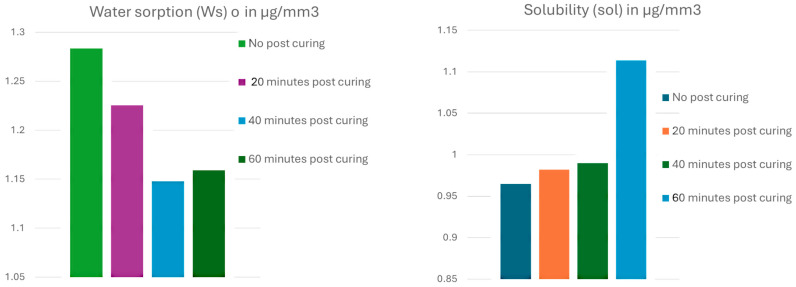
Visual presentation of different post-curing times and the mean water sorption (Wso) and solubility (Sol) of 3D-printed resin composite.

**Figure 6:**
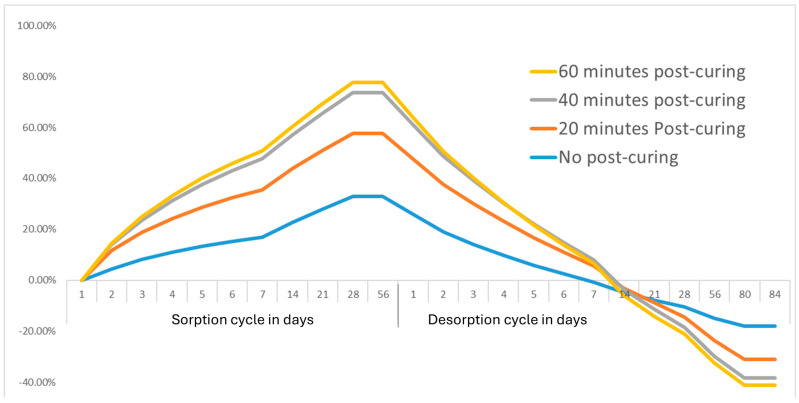
Percentage mass changes during water sorption and subsequent desorption cycles of 3D-printed composite resin specimens subjected to different post-curing durations (0, 20, 40, and 60 min). Sorption was measured for 56 days, followed by a desorption period of 84 days. Initially, higher post-curing durations demonstrated increased relative water uptake; however, these groups subsequently showed superior water desorption, ultimately resulting in lower equilibrium moisture content compared to the non-post-cured group.

The normality of distribution was confirmed, using the Shapiro–Wilk test, for water sorption (*p* < 0.003), and solubility (*p* < 0.01). A robust analysis examining the homogeneity of variances revealed significant differences in the variance of water sorption among the post-curing groups (F(3,16) = 5.43, *p* = 0.009), and in solubility among the groups (F(3,16) = 5.15, *p* = 0.01), thus violating the assumption of equal variances required for standard ANOVA. Therefore, an adjusted Welch’s ANOVA, which does not require the assumption of equal variances, was performed. The post-printing polymerization time significantly influenced the water sorption and solubility of the material (*p* < 0.01). Table 4 summarizes the absolute values for water sorption (Wso) and solubility (Sol) measured after standardized 2-month sorption and desorption cycles. The non-post-cured group exhibited the highest absolute water sorption (1.28 ± 0.12 mg/mm^3^). Increasing post-curing duration significantly reduced absolute water uptake, with the 40 min (1.14 ± 0.15 mg/mm^3^) and 60 min (1.15 ± 0.05 mg/mm^3^) groups demonstrating statistically lower sorption values (Figure 5). Regarding solubility, extending the post-curing durations slightly increased the solubility values; they reached their highest in the 60 min group (1.11 ± 0.07 mg/mm^3^), compared to the lowest solubility, which was recorded in the non-cured group (0.96 ± 0.09 mg/mm^3^). Figure 6 illustrates the percentage of mass change over the water sorption (56 days) and subsequent desorption (84 days) cycles for samples subjected to different post-curing durations. Initially, during the sorption phase, samples with longer post-curing durations (40 and 60 min) showed higher initial relative mass gains, reaching peaks of approximately 60–75%, compared to the lower peaks (~20%) observed in the no-post-curing samples. However, in the subsequent desorption phase, these same samples exhibited a more effective release of absorbed water, with the mass decreasing notably below initial baseline levels, indicating superior water desorption capacity. The non-cured group retained a higher residual moisture content above the baseline. Taken together, these results show that extended post-curing durations initially facilitate a more dynamic water interaction and a higher relative initial sorption, followed by significantly improved desorption, and leading ultimately to reduced equilibrium water sorption values, despite a slight increase in solubility. This indicates an observation of overall enhancement in the moisture resistance of the composite resin with 40 min of post-curing.

## 4. Discussion

The current analysis investigated how post-printing polymerization time affects monomer release and water sorption. The present results highlight the significant impacts of post-curing time and storage conditions on the release of BisEMA monomers from the 3D-printed dental composites and their water sorption and solubility behaviors. Therefore, the null hypothesis was rejected.

The four main common dental monomers (BisEMA, BisGMA, UDMA, and TEGDMA) were separated and calibrated using HPLC to ensure a comprehensive analysis of the tested samples. BisEMA was detected in the samples; other monomers were not present. This observation could be due to their absence in the resin formulation or the monomer concentration values falling below the detectable limits. A thorough analysis, which involved calibration of all four primary dental monomers, was undertaken to validate the manufacturer’s safety data sheet [20], rule out the possibility of undisclosed monomers, and confirm that BisEMA was the predominant base monomer. Multiple recent analyses confirm that Bisphenol A ethoxylated dimethacrylate (Bis-EMA) is often the primary (and sometimes sole detectable) base monomer in certain 3D-printed dental resins, with little to no Bis-GMA, UDMA, or TEGDMA present. For example, a recent study by Penzenstadler et al. used LC–MS/MS to profile four different 3D-printed denture/base resins. It found that in one resin sample (Saremco’s CrownTec denture base material), Bis-EMA accounted for >80% of the total unpolymerized methacrylate content, whereas other common monomers were minimal or undetected [21]. In contrast, Berghaus et al. detected eluted BisGMA, TEGDMA, and BisEMA within their experimental formulation. Their analysis included residual monomers from experimental 3D composites over 10 days, the monomer matrix of which was composed of 53 wt.% BisEMA, 35 wt.% BisGMA, and 12 wt.% TEGDMA [15].

The present in vitro study used the HPLC evaluation method, which allows for quantitative and qualitative analyses of components contained in a sample and the determination of how much of each component is present [22]; this is the currently recommended method for the analysis of leached monomers from dental resins. BisEMA exhibited a relative tendency toward elution, which could be attributed to the absence of hydrogen bond donor groups, such as OH, in its structure [23] and the presence of hydrophobic aromatic rings in its chemical formula [24]. Unlike other monomers, which form hydrogen bonds, BisEMA lacks this feature, and this contributes to its lower intermolecular forces and network stability [25]. BisEMA is a hydrophobic monomer whose structure (aromatic bisphenol A core with ethoxylated aliphatic linkages) lacks any hydroxyl groups to donate hydrogen bonds [26]. As a result, BisEMA cannot strongly participate in water’s hydrogen-bonding network; it interacts with water only via relatively weak hydrogen-bond acceptor sites (ether or carbonyl oxygens) and non-polar van der Waals forces [27]. In contrast, ethanol (a less polar protic solvent) can penetrate a BisEMA-based resin more effectively because ethanol’s –OH groups can form hydrogen bonds with BisEMA’s oxygen atoms while its ethyl backbone interacts with BisEMA’s hydrophobic aromatic/aliphatic regions. Consequently, BisEMA-containing resins tend to absorb significantly more ethanol than water, and replacing hydroxyl-bearing monomers (like Bis-GMA) with BisEMA markedly reduces water sorption. Therefore, when the BisEMA polymer network is exposed to external media, such as water or ethanol, the absence of hydrogen bonds makes it easier for the media to penetrate and disrupt the network. This disruption facilitates the release of residual, unreacted BisEMA monomers that may have remained trapped within the structure after polymerization [2,28]. Moreover, aromatic rings act as barriers preventing water molecules from breaking down the BisEMA network, rendering BisEMA more resistant to elution in aqueous media than in ethanol. The aromatic (benzene) rings also contribute to the characteristic water sorption behaviors of Bis-EMA polymers [7,29].

Bisphenol A (BPA) has attracted considerable attention in the context of dental resin materials, even though BPA itself is typically not a formal ingredient in modern composites. Instead, BPA is relevant as the structural precursor for several important dental monomers. For instance, Bis-GMA and Bis-EMA are both derivatives synthesized from BPA. During the manufacture of these monomers, trace residual BPA may remain as an impurity and subsequently be present in the final resin material at extremely low levels [30,31]. A balanced assessment of BPA in dental composites recognizes that, while the presence of any endocrine disruptor is undesirable, the actual exposure levels from dental treatment are extremely low, and exposures are brief. Recent systematic reviews and meta-analyses have reinforced that BPA release from resin-based dental materials is transient and decreases rapidly with time, especially if clinicians follow best practices such as curing properly [3,32]. Contemporary evidence indicates that the cytotoxicity of dental resin composites, including newer 3D-printed materials, is principally driven by leachable monomers and photoinitiator fragments, whereas BPA contributes a minor portion of exposure as a byproduct. The regulatory and scientific consensus, supported by the foundational literature and up-to-date research, holds that BPA exposure from these restoratives is far below harmful levels. Nonetheless, the dental research community continues to refine resin formulations (e.g., developing BPA-free derivatives and more reactive photoinitiators) and to implement clinical protocols that further minimize any potential risks, thereby ensuring that these widely used restorative materials remain safe and biocompatible in the long term [33,34,35].

The water sorption and solubility results described in this study further illustrate the importance of post-curing time in influencing the interaction of the material with water. The stability of the 40 min post-curing group during the water sorption/desorption cycles indicates that this post-curing duration also provides optimal resistance to water absorption and desorption, which is critical for maintaining the dimensional stability and longevity of dental restorations [10].

Extending the post-curing time beyond 40 min did not lead to a significant further reduction in monomer elution or water sorption. This finding is likely due to several factors, which might include 3D printing technology, limited monomer diffusion, and the interaction of fillers with the resin composition. In the experimental setup, 3D printing was performed with a DLP printer (Digital Light Processing). DLP technology uses a digital projector to selectively cure entire layers of resin by exposing them to UV light. Each layer is fully cured before the next is printed, resulting in a high degree of polymerization within each layer [36]. As DLP cures entire layers rather than individual points (as in stereolithography) [37], this initial layer-by-layer curing is inherently highly effective in achieving optimal post-printing polymerization within a shorter period [38]. Further, during 3D printing, not all monomers react and link together to form the final, hardened shape. Some remain unreacted and entrapped within the printed parts [39,40]. For these unreacted monomers to participate in further curing during post-printing polymerization, they need to reach the surface. However, the complex internal structure of the 3D-printed parts can create obstacles and long pathways, making it difficult for these monomers to move or diffuse to the surface [41]. After a certain point of time (e.g., the one observed at 40 min), the diffusion of monomers from deeper within the structure to the surface slows down, and further curing does not significantly impact the monomer release rate [41,42].

In addition, the storage conditions played a significant role in the concentration of monomer release, with ethanol showing a more aggressive impact than water, particularly over longer durations. This finding aligns with the existing literature on residual monomers, describing barely detectable amounts in water, compared with noticeably high elution in ethanol [15,43,44].

While 40 min appears to be an effective post-curing duration within the current experimental setup, it is important to acknowledge the limited generalizability of this finding, as the optimal post-curing time significantly varies depending on other post-curing settings, such as irradiance and intensity [45].

## 5. Conclusions

Within the limitations of the present study, the following findings were reported:

- Forty minutes of post-curing was the most effective duration for minimizing the release of residual monomers, particularly BisEMA, and reducing water sorption.
- Prolonged post-curing beyond 40 min did not result in further improvements.
- The storage medium significantly impacted monomer release, with ethanol demonstrating higher aggressiveness than water.

These findings emphasize the need to carefully implement appropriate post-curing protocols to enhance the performance and longevity of 3D-printed indirect restorations. Future studies should incorporate an assessment of the degree of conversion to better understand its correlation with monomer elution and water sorption behaviors.

## Figures and Tables

**Table 1:** Details as to the 3D printing in the experimental design.

Material	Manufacturer (Company)	Resin Composition	3D Printer
Permanent Crown Shade A3 (Lot Number 600164)	Formlabs Inc., Somerville, MA, USA.	Inorganic fillers: Ceramic micro-filler Organic Polymers: Bis-EMA Photoinitiator system: TPO, photoinitiator	Pionext D128, Piocreat 3d, Shenzhen, China.

**Table 2:** Information on the reference standards for the monomers used in this study.

Reference Substance (Abbreviation)	Chemical Nomenclature	Function	CAS# *	Manufacturer	Product Number
BisGMA	Bisphenol A glycerolate dimethacrylate	Monomer	1565-94-2	Sigma-Aldrich, St. Louis, MO, USA	494356
BisEMA	Bisphenol A ethoxylate dimethacrylate	Monomer	41637-38-1	Sigma-Aldrich, St. Louis, MO, USA	455059
UDMA	Urethane-di-methacrylate	Co-Monomer	72869-86-4	Sigma-Aldrich, St. Louis, MO, USA	436909
TEGDMA	Triethylenglycol-dimethacrylate	Co-Monomer	109-16-0	Sigma-Aldrich, St. Louis, MO, USA	261548

CAS# *: Chemical Abstracts Service: A registry number to provide an identifier for chemical substances.

**Table 3:** Results of kernel regression model for monomer release, reported in µg/mL.

Effect	Comparison	Crude Analysis	SE	z-Value	*p*-Value	95% Confidence Interval
Mean Concentration		425.3676	59.75953	7.12	<0.001	[304.0363, 548.1826]
Effect of post-curing time	20 min post-curing vs. no post-curing	−478.95	80.15424	−5.98	<0.001	[−630.0968, −336.0107]
	40 min post-curing vs. no post-curing	−519.963	80.8881	−6.43	<0.001	[−661.6359, −365.1328]
	60 min post-curing vs. no post-curing	−480.1584	78.91344	−6.08	<0.001	[−630.2413, −333.1834]
Effect of storage condition	H_2_O 7 d vs. H_2_O 24 h	0.27259	19.15515	0.01	0.989	[−8.618262, 15.89887]
	ethanol 24 h vs. water 24 h	754.668	68.29158	11.05	<0.001	[608.6126, 886.7524]
	ethanol 7 d vs. water 24 h	872.4947	66.50705	13.12	<0.001	[729.3719, 988.1243]

**Table 4:** Water sorption (Wso), and solubility (Sol) among the groups in the study after 2 months of water sorption cycle and 2 months of desorption cycle (solubility).

Post-Curing Duration	Average Wso (μg/mm^3^)	Average Sol (μg/mm^3^)
No post-curing	1.28 ± 0.12 ^a^	0.96 ± 0.09 ^a^
20 min post-curing	1.22 ± 0.21 ^b^	0.98 ± 0.14 ^b^
40 min post-curing	1.14 ± 0.15 ^ab^	0.99 ± 0.12 ^ab^
60 min post-curing	1.15 ± 0.05 ^a^	1.11 ± 0.07 ^a^

Values sharing the same superscript letters within a column have no statistically significant difference (*p* > 0.05), while values with different letters have statistically significant differences (*p* < 0.05).

## Data Availability

The original contributions presented in this study are included in the article. Further inquiries can be directed to the corresponding author.

## Abbreviations

The following abbreviations are used in this manuscript:

Wso	Water sorption
Sol	Solubility
HPLC	High-performance liquid chromatography
